# Relationship between feed efficiency and gut microbiota in laying chickens under contrasting feeding conditions

**DOI:** 10.1038/s41598-024-58374-3

**Published:** 2024-04-08

**Authors:** Maria Bernard, Alexandre Lecoeur, Jean-Luc Coville, Nicolas Bruneau, Deborah Jardet, Sandrine Lagarrigue, Annabelle Meynadier, Fanny Calenge, Géraldine Pascal, Tatiana Zerjal

**Affiliations:** 1 INRAE, AgroParisTech, GABI, Université Paris-Saclay, 78350 Jouy-en-Josas, France; 2 INRAE, SIGENAE, 78350 Jouy-en-Josas, France; 3 INRAE, INSTITUT AGRO, PEGASE UMR 1348, Saint-Gilles, France; 4 GenPhySE, Université de Toulouse, INRAE, ENVT, 31326 Castanet-Tolosan, France

**Keywords:** Microbiome, Animal breeding

## Abstract

The gut microbiota is known to play an important role in energy harvest and is likely to affect feed efficiency. In this study, we used 16S metabarcoding sequencing to analyse the caecal microbiota of laying hens from feed-efficient and non-efficient lines obtained by divergent selection for residual feed intake. The two lines were fed either a commercial wheat-soybean based diet (CTR) or a low-energy, high-fibre corn-sunflower diet (LE). The analysis revealed a significant line x diet interaction, highlighting distinct differences in microbial community composition between the two lines when hens were fed the CTR diet, and more muted differences when hens were fed the LE diet. Our results are consistent with the hypothesis that a richer and more diverse microbiota may play a role in enhancing feed efficiency, albeit in a diet-dependent manner. The taxonomic differences observed in the microbial composition seem to correlate with alterations in starch and fibre digestion as well as in the production of short-chain fatty acids. As a result, we hypothesise that efficient hens are able to optimise nutrient absorption through the activity of fibrolytic bacteria such as *Alistipes* or *Anaerosporobacter*, which, via their production of propionate, influence various aspects of host metabolism.

## Introduction

Chicken eggs are consumed worldwide and remain one of the most cost-effective animal-based sources of nutrition in the human diet. According to the OECD-FAO, global egg production has doubled since 1990 and is projected to increase by another 15% from 2021 to 2031^1^. In the chicken industry, feeding costs account for up to 70% of the total production cost^2^, making feed efficiency a crucial phenotype for reducing production expenses and minimising feed waste and pollutants.

Feed efficiency measures the ability of an animal to make the most of its feed ration for maintenance and production, and in poultry, it is commonly evaluated using two indices. The feed conversion ratio (FCR) in layers measures the amount of feed required to produce one kilogram of eggs, while the residual feed intake (RFI) is the difference between an animal’s observed feed intake (FI) and its predicted FI, which is statistically estimated based on maintenance and production requirements. RFI is considered a better proxy of feed efficiency than FCR as its estimation takes into account variations in maintenance energy expenditure^3^.

Feed efficiency is a complex trait that is partly shaped by genetics^4^, and selective efforts in chickens have targeted either modifications to the metabolism of birds^5^ or their digestive capacity^6^. Environmental factors such as rearing conditions and diet composition also have an influence^7^. This is particularly significant given the globalisation of poultry production, which means that animals are exposed to a wide range of environments and feeding regimes. Recent research has highlighted the role of the gut microbiota in variations in digestive ability, and there is growing evidence that it can contribute to feed efficiency.

Indeed, the gut microbiota is known to be involved in a number of important processes, including immunity, behaviour, and digestion of feed for energy harvesting by the host^8,9^. The microbial communities present in the different compartments of the intestine enable the degradation of feed that is not digested by host enzymes^8^. Bacterial richness and diversity have been found to be particularly high in the caecum, where transit time is the longest compared to other intestinal segments^10,11^ and major nutrients influencing the host metabolism^7,8,12,13^—such as vitamins B and K and short-chain fatty acids (SCFA)—are produced. The gut ecosystem evolves throughout the host’s life and is particularly sensitive to various factors such as the genetics of the host, the rearing conditions, and feed formula^12,14^.

Many studies have investigated the association between microbiota composition and feed efficiency in chickens^10,11,15–19^, but the findings have been inconsistent. For example, Siegerstetter et al.^17^ found reduced richness and diversity in the caecal microbiota of efficient broilers (based on RFI), whereas Stanley et al.^16^ reported a richer microbiota in efficient broilers (based on FCR) but with similar diversity. Conversely, Yan et al.^11^ found no difference in microbial diversity between poorly and highly efficient layers (based on RFI). These disparate findings may be explained by the extensive physiological and genetic differences between layers and broilers^12,20,21^. Information from the literature focuses mainly on broiler chickens and very little on layers^12,21^, making it difficult to compare microbiota studies. The microbiota is also highly sensitive to environmental changes, so much so that even studies with multiple replicates under similar experimental conditions have not always achieved concordance among trials. Indeed, significant differences in richness and diversity were observed in one of the three trials of Stanley et al.^16^, and in one of two geographical locations of Siegerstetter et al.^17^. Additionally, the lack of agreement among studies may also result from the limited ability to separate animals from standard populations based on their efficiency, as even the extremes of the feed efficiency distribution differ by relatively little^11,16,17^.

In this study, we aimed to analyse the microbiota of adult laying hens to explore its contribution to chicken feed efficiency and investigate its sensitivity to feed changes. Specifically, we used a 16S rRNA gene metabarcoding approach to characterise the caecal microbiota of two RFI-divergent laying lines (R+ and R−) that are the result of more than 40 years of selection^22^. The advantage of using these lines is that, despite sharing a similar genetic background, their RFI values differ by more than five phenotypic standard deviations^3^. The two lines have comparable egg production and growth rates. However, thanks to this long-running programme of selection, they differ considerably in feed intake (56% higher in R+ than in R− hens) and other RFI-related traits such as fat deposition (higher in R− chickens) and diet-induced thermogenesis (higher in R+ chickens), indicating strong differences in energy metabolism between lines^3,23–25^. Hens were fed two diets, one representing the classical layer commercial diet (control, CTR) and the other a fibre-rich but energy-depleted diet (low-energy, LE) that might be found in countries with difficulties in accessing certain resources. This experimental design allowed us to (i) investigate the link between feed efficiency and the caecal microbiota, and (ii) explore the stability of this potential link under different feeding conditions.

## Results

### Line and diet effects on egg production and efficiency-related traits

Feed intake, feed efficiency indices (RFI and FCR), growth, and egg production traits were recorded for hens of the high efficient (R−) and the non-efficient (R+) lines fed either an optimal diet (CTR) or a low-energy, high-fibre diet (LE) as outlined in Table 1. Notably, significant differences in feed intake (FI) and efficiency indices were observed between the lines across both dietary conditions. Specifically, under the LE diet, the energy intake decreased by a 9% for the R+ and by a 15% for the R− lines compared to the CTR diet, despite no significant difference in FI between the two diets. LE-fed hens presented also reduced egg weights and yolk weights. However, no significant line or diet effects were observed for the other traits measured.

**Table 1:** Least square means (± standard error) and significance estimates for line and diet effects on efficiency and production measures.

Measures	R− CTR	R+ CTR	R− LE	R+ LE	ANOVA *P*-values	
					Line	Diet
Feed Intake, FI (kg/28 d)	2.6 ± 0.1	4.0 ± 0.1	2.7 ± 0.1	4.1 ± 0.1	< 0.001*	0.390
Energy Intake (MJ/28 d)	29.4 ± 1.1	45.2 ± 1.1	25.2 ± 1.3	41.0 ± 1.3	< 0.001*	0.005*
Residual Feed Intake, RFI (kg/28 d)	− 0.6 ± 0.1	0.8 ± 0.1	− 0.3 ± 0.1	1.1 ± 0.1	< 0.001*	0.001*
Feed Conversion Rate, FCR (kg /28 d)	2.3 ± 0.1	3.6 ± 0.1	2.6 ± 0.1	3.8 ± 0.1	< 0.001*	0.039*
Egg laying rate (%)	86.6 ± 1.8	86.6 ± 1.8	84.9 ± 2.2	84.9 ± 2.2	1.000	0.475
Egg number	59.9 ± 1.9	59.9 ± 1.9	60.3 ± 2.2	60.3 ± 2.3	0.991	0.862
Egg weight (g)	48.6 ± 0.5	47.7 ± 0.5	47.2 ± 0.6	46.3 ± 0.6	0.176	0.050*
Egg mass (kg/28 d)	1.1 ± 0.0	1.1 ± 0.0	1.1 ± 0.0	1.1 ± 0.0	0.253	0.439
Yolk weight (g)	13.7 ± 0.2	13.4 ± 0.2	13.3 ± 0.2	13.0 ± 0.2	0.058	0.026*
Yolk proportion (%)	27.6 ± 0.3	27.8 ± 0.3	27.4 ± 0.3	27.6 ± 0.3	0.054	0.667
Body weight (kg)	2.0 ± 0.1	2.1 ± 0.1	1.9 ± 0.1	2.0 ± 0.1	0.058	0.071

R− and R+ are the efficient and non-efficient lines, respectively, and CTR and LE are the control and low-energy diets, respectively. Significant effects (*P*-value ≤ 0.05) are highlighted by an asterisk. The line x diet interaction was not significant and was thus removed from the model.

### Microbiota composition, richness and diversity analyses

The 16S metabarcoding sequencing generated 3,077,915 paired-end reads, with between 25,783 and 78,980 read pairs per sample. From these sequences, we obtained 609 clusters as amplicon sequence variants (ASV) and eight of these were removed because of their weak prevalence. After processing, we obtained 601 ASVs, and each sample contained an average of 24,505 sequences. At the species level, 79.4% of the ASVs were affiliated to “unknown species” and 9.8% to multiple species. At the genus level, 39.4% were affiliated to “unknown genus” and 3.0% had ambiguous taxonomy. Considering the uninformative nature of most of the species-level affiliations, we used genus affiliations in all subsequent taxonomic analyses.

The two dominant phyla were *Bacteroidota*, and *Firmicutes* (Table 2). Within *Bacteroidota*, the most abundant families were *Bacteroidaceae* (36.6%), *Barnesiellaceae* (9.1%), and *Rikenellaceae* (4.7%), while the most abundant families of *Firmicutes* were *Ruminococcaceae* (23%) and *Lachnospiraceae* (7.7%). Proportions of *Firmicutes* were rather similar among the different line x diet groups. Instead, the relative abundance of *Bacteroidota* tended to be lower in the R+ line than in R− under both diets.

**Table 2:** Phylum relative abundance (± standard error) and significance estimates for line and diet effects.

Phylum	Nb of ASV	Mean ± SE	R− CTR	R+ CTR	R− LE	R+ LE	ANOVA *P*-values	
							Line	Diet
Bacteroidota	84	50.4 ± 0.2	53.1 ± 2.8	46.4 ± 2.7	54.9 ± 3.2	48.2 ± 3.2	0.052	0.613
Firmicutes	500	45.1 ± 0.2	42.8 ± 2.4	46.7 ± 2.3	43.5 ± 2.8	47.4 ± 2.8	0.186	0.828
Actinobacteriota	9	1.9 ± 0.4	1.8 ± 0.8	3.9 ± 0.7	0.0 ± 0.9	1.4 ± 0.9	0.027*	0.010*
Campylobacterota	2	1.4 ± 0.2	0.8 ± 0.4	1.4 ± 0.4	1.6 ± 0.5	2.2 ± 0.5	0.246	0.110
Proteobacteria	3	0.8 ± 0.1	1.1 ± 0.2	1.2 ± 0.2	0.3 ± 0.2	0.4 ± 0.2	0.549	0.000*
Desulfobacterota	3	0.4 ± 0.0	0.4 ± 0.0	0.4 ± 0.0	0.5 ± 0.1	0.5 ± 0.1	0.987	0.152

This table indicates the total number of ASVs per phylum, the global mean abundance, the least square mean abundance in each line x diet group ± standard error (SE), and the *P*-values for the effect of line (R− vs. R+) or diet (CTR for control vs. LE for low-energy) on phylum relative abundance. Significant effects (*P*-value ≤ 0.05) are highlighted by an asterisk. Line x diet interaction was not significant and was thus removed from the model.

The *Actinobacteriota* were significantly influenced by line, being more abundant in the R+ line compared with the R− line, and by diet, being more abundant under the CTR diet compared with the LE diet. Finally, the *Proteobacterota* were more abundant in hens fed the CTR diet compared to those fed the LE diet.

**Figure 1:**
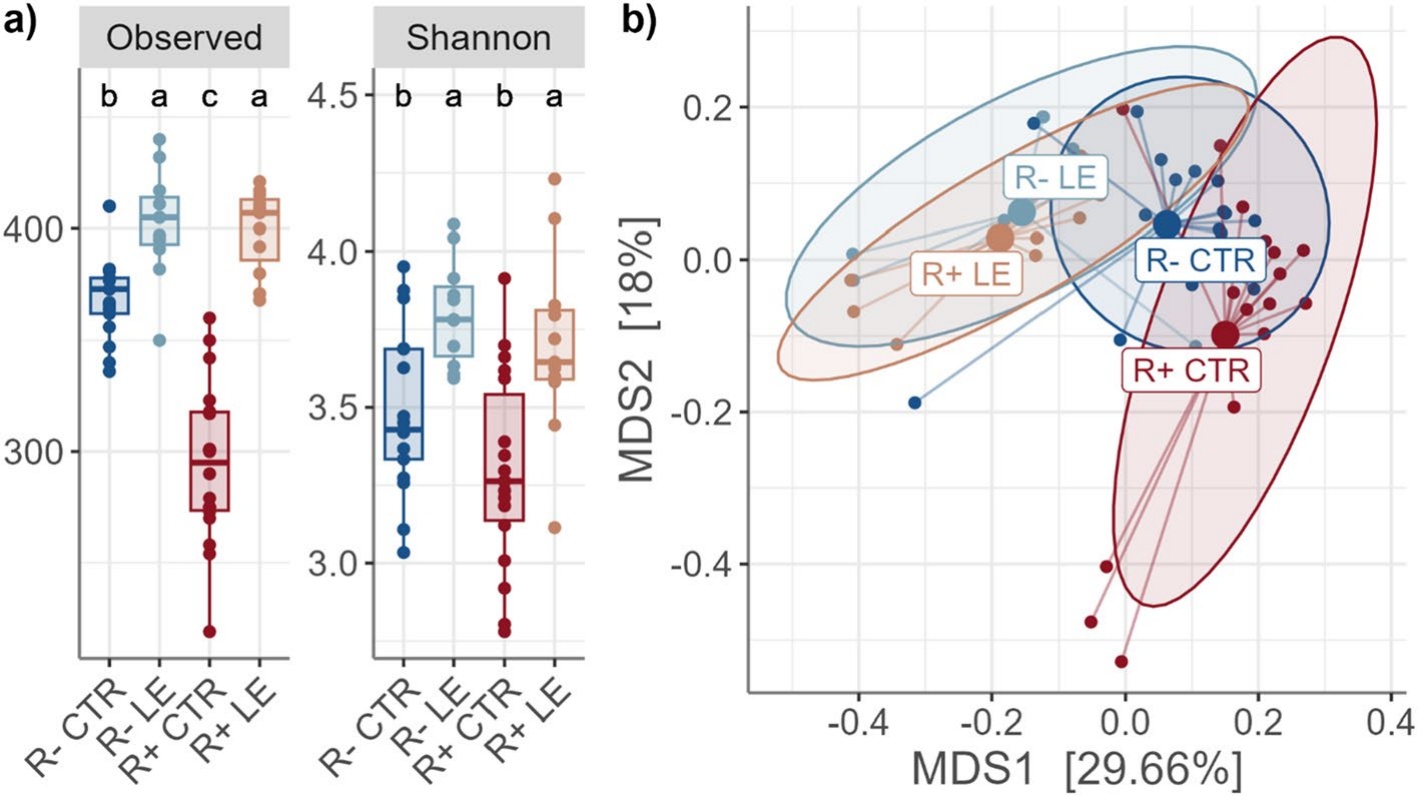
Alpha diversity distribution and beta diversity ordination plot: (**a**) Alpha diversity indices: observed richness and Shannon. In each panel, different letters at the top indicate significant pairwise differences (*P*-value ≤ 0.05) between line x diet groups. (**b**) Multidimensionnal scaling (MDS) of Bray Curtis dissimilarity between line x diet groups. Colours represent the four line x diet groups: red = R+ line fed the CTR diet; dark blue = R− line fed the CTR diet; orange = R+ line fed the LE diet; light blue = R− line fed the LE diet.

The richness and diversity analyses revealed a significant line and diet effect on the microbiota (Fig. 1). Interestingly, the diet effect was milder in the R− line compared to the R+ line. More specifically, the change from the CTR to the LE diet induced a significant increase in richness in both lines but the range of change was not the same between lines, + 8% for the R− line and +35% for the R+ line. This explained the significant line x diet interaction observed (*P*-value < 0.001). The Shannon index values were also affected by line (*P*-value = 0.034) and diet (*P*-value < 0.001) (Fig. 1a). This trend was also evident in the multidimensional scaling (MDS) analysis of beta diversity (Fig. 1b), where distinct clustering patterns were observed. Specifically, the R− LE and R+ LE groups were clearly separated from the R+ CTR group, while the R− CTR group occupied an intermediary position. Statistical analyses validated these findings, confirming significant effects for both line (*P-*value = 0.007) and diet (*P*-value < 0.001).

### Identification of taxonomic and functional differences in microbiota

The differential abundance analysis allowed us to identify the ASVs contributing to the differences between line x diet groups. The four comparison groups consisted of: (i) R+ versus R− hens fed the CTR diet, (ii) R+ versus R− hens fed the LE diet, (iii) R + hens fed the LE diet versus the R+ hens fed the CTR diet, and (iv) R− hens fed the LE diet versus the R− hens fed the CTR diet. Supplementary Table S1 provides the full results for the 94 Differential Abundant (DA) ASV in at least one of the four comparisons.

When examining the diet effect (LE versus CTR), we observed a substantial discrepancy in the number of DA ASVs between the R+ and R− lines. Specifically, the R+ line presented the highest number of DA ASVs (80), whereas the effect was smaller in the R− line (13). On the other hand, when analysing the line effect (R+ versus R−), we observed the highest number of DA ASVs under the CTR diet (39), whereas the effect was minimal under the LE diet (1 DA ASVs). These results reinforced the pattern observed previously highlighting the impact of the diet in both lines, albeit to a lesser extent in the R- line, and a line effect particularly evident in the CTR diet.

**Figure 2:**
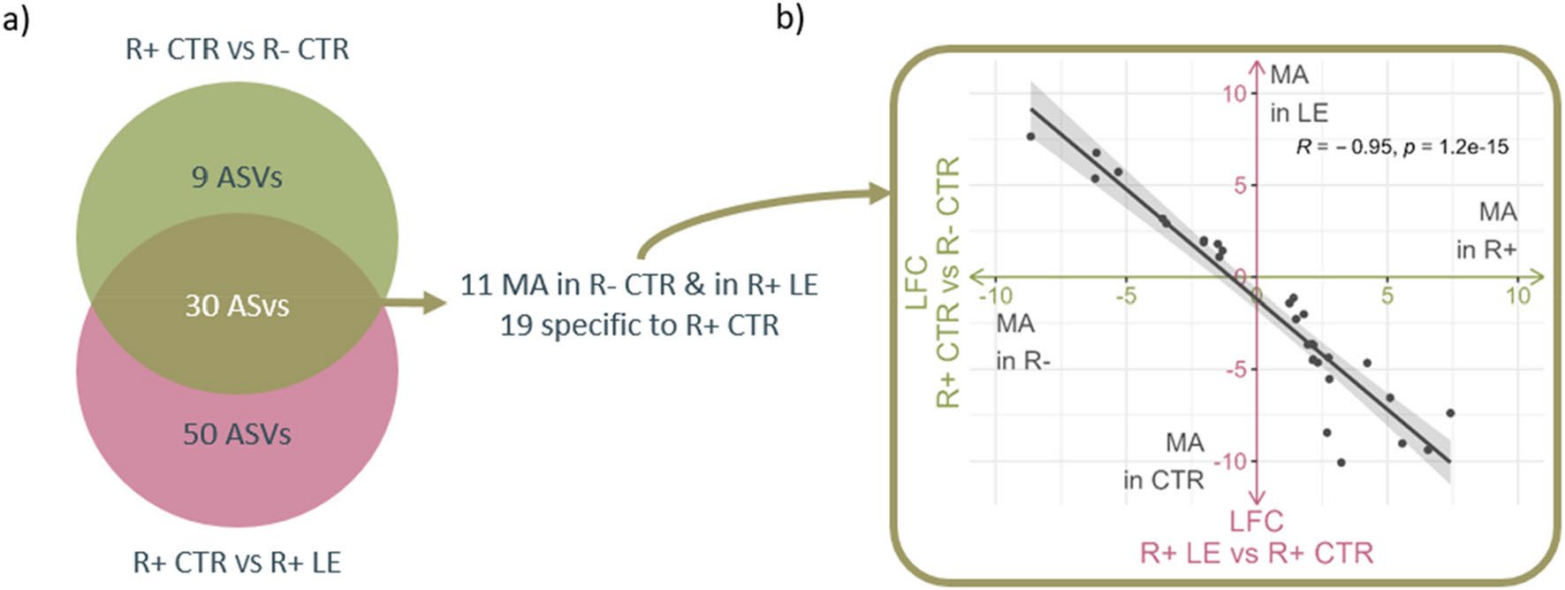
Venn diagram and log fold change of differentially abundant (DA) ASVs. (**a**) Venn diagram depicting unique and shared DA ASV between those identified from the R+ CTR versus R− CTR comparison and those identified from the R+ LE versus R+ CTR comparison; (**b**) Log-fold change (LFC) of shared DA ASVs, represented with black dots, between the two comparisons. Eleven ASVs that were more abundant (MA) in the R− fed CTR diet were also more abundant in the R+ fed the LE (top left corner of the plot). Conversely, 19 ASVs were specific to the R+ CTR group (bottom right corner of the plot).

By investigating ASVs affected by both line and diet, we could identify ASVs that were shared between groups and others that were specific to one group. Interestingly, we identified 30 ASVs that were DA both between the R+ CTR versus R- CTR, and between the R+ LE versus R+ CTR (Fig. 2a). Among these, 11 ASVs presented higher abundance in R- CTR compared with R+ CTR and in R+ LE compared with R+ CTR. This displayed that ASVs that were more abundant in the R- line compared with the R+ line under standard conditions (CTR) become more abundant in the R+ hens when fed the LE diet (Fig. 2b). Conversely, the remaining 19 DA ASV were, specific to the R+ CTR group.

### Distribution of bacterial taxonomies among line and diet comparisons

**Figure 3:**
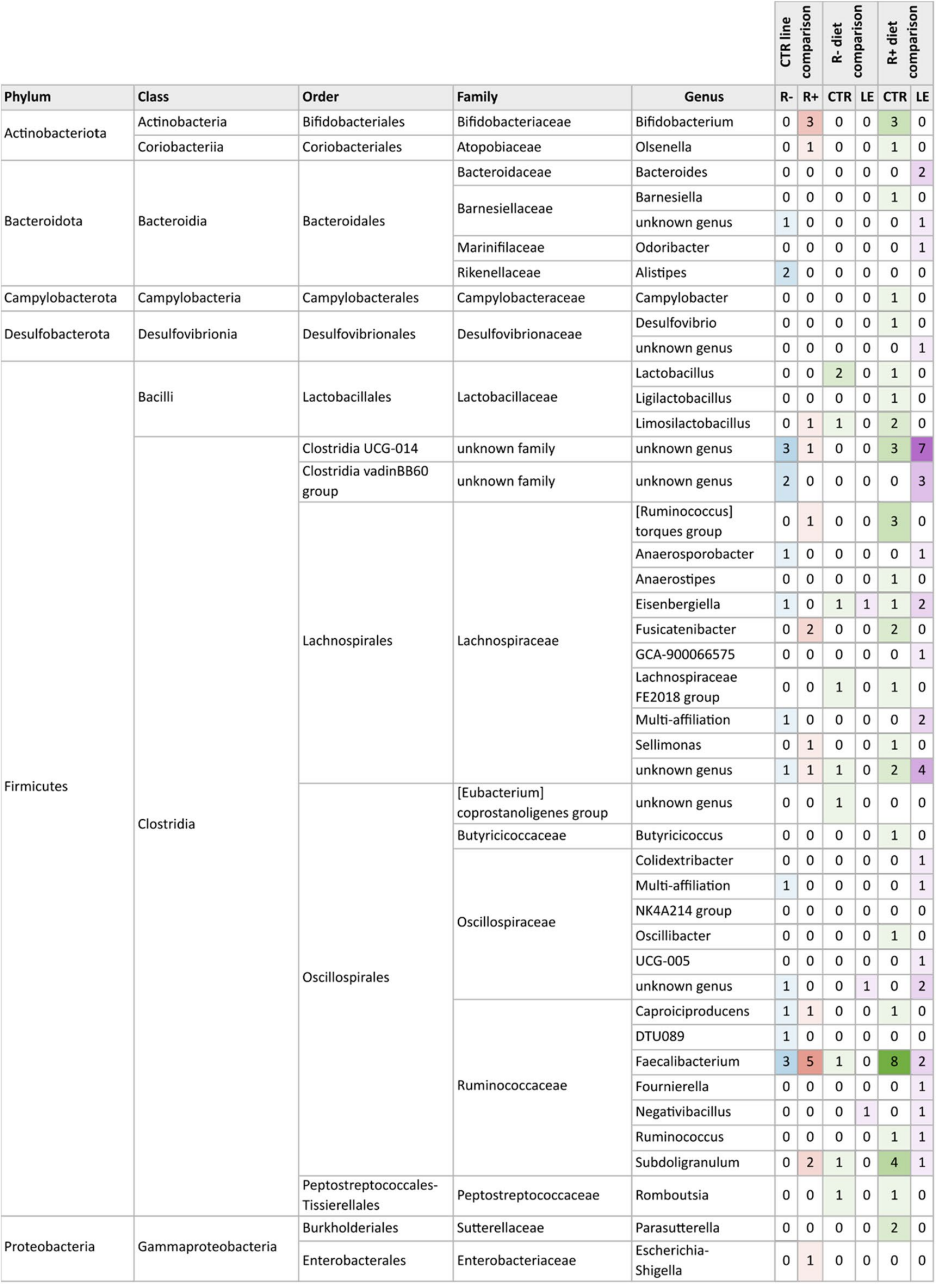
Heatmap illustrating the number of ASVs significantly more abundant in one group within each comparison. The CTR line comparison displays ASVs identified as differentially abundant in the R+ CTR group (depicted in red) versus the R− CTR group (depicted in blue). The R− and R+ diet comparisons reports ASVs differentially abundant in the R− LE group (depicted in purple) versus the R− CTR group (depicted in green), or in the R+ LE (depicted in purple) versus R+ CTR (depicted in green).

In terms of taxonomic distribution, the vast majority of DA ASVs belonged to the phylum of *Firmicutes* (77 ASVs), with a significant portion (93%) assigned to the order *Clostridia*. The remaining 17 DA ASVs were distributed across *Bacteroidota* (7%), *Actinobacteriota* (4%), *Proteobacteria* (3%), *Desulfobacterota* (2%), and *Campylobacterota* (1%). Taxonomic assignments are presented in Fig. 3 and detailed in Supplementary Table S1.

Many taxonomic groups exhibiteda pattern similar to that described for the DA ASVs, highlighting the distinctiveness of the R+ CTR group from the other groups. Some taxa were associated with DA ASVs specifically associated with the R+ CTR group, and conversely, others showed higher abundace in the R- CTR and in R+ LE groups. In particular, DA ASVs associated with the phylum *Actinobacteriota* were more abundant in the R+ CTR group and three of these, from the *Bifidobacterium* and *Olsenella* genera, presented mean relative abundances between 0.5 and 3%. Conversely, the DA ASVs associated with phylum *Bacteroidota* were generally more abundant in the R- CTR group (e.g., genus *Alistipes*) or in the R+ LE group (e.g., genus *Bacteroides*). Notably, the change in abundance of DA ASV affiliated to the latter was remarkable and passed from a relative abundance of 4.3% in the R+ CTR to 18.2% in the R+ LE group. Other taxa were associated to ASVs that were differential in several groups. For example, the genus *Faecalibacterium* was associated with three DA ASVs that were more abundant in the R- CTR group and five DA ASVs that were more abundant in the R+ CTR group. The comparison between the CTR diet and the LE diet also revealed abundance changes in several other genera belonging to the families *Lactobacillaceae*, *Lachnospiraceae*, or *Ruminococcaceae*.

### Comparison of inferred microbial functions

KEGG functional inference allowed to identify 4,403 functions. Differential function analysis revealed that the greatest function shift occurred between the R+ CTR and the R+ LE groups, presenting 841 DA functions. In contrast, the change between CTR to LE diets in the R- line was less dramatic, with 217 DA functions identified. The functional differences between R+ and R− lines were mainly observed under the CTR diet, with 329 DA functions identified. Conversely, under the LE diet, the functional profiles of the two lines showed a high degree of similarity, with only 43 DA functions detected (Supplementary Table S2).

**Figure 4:**
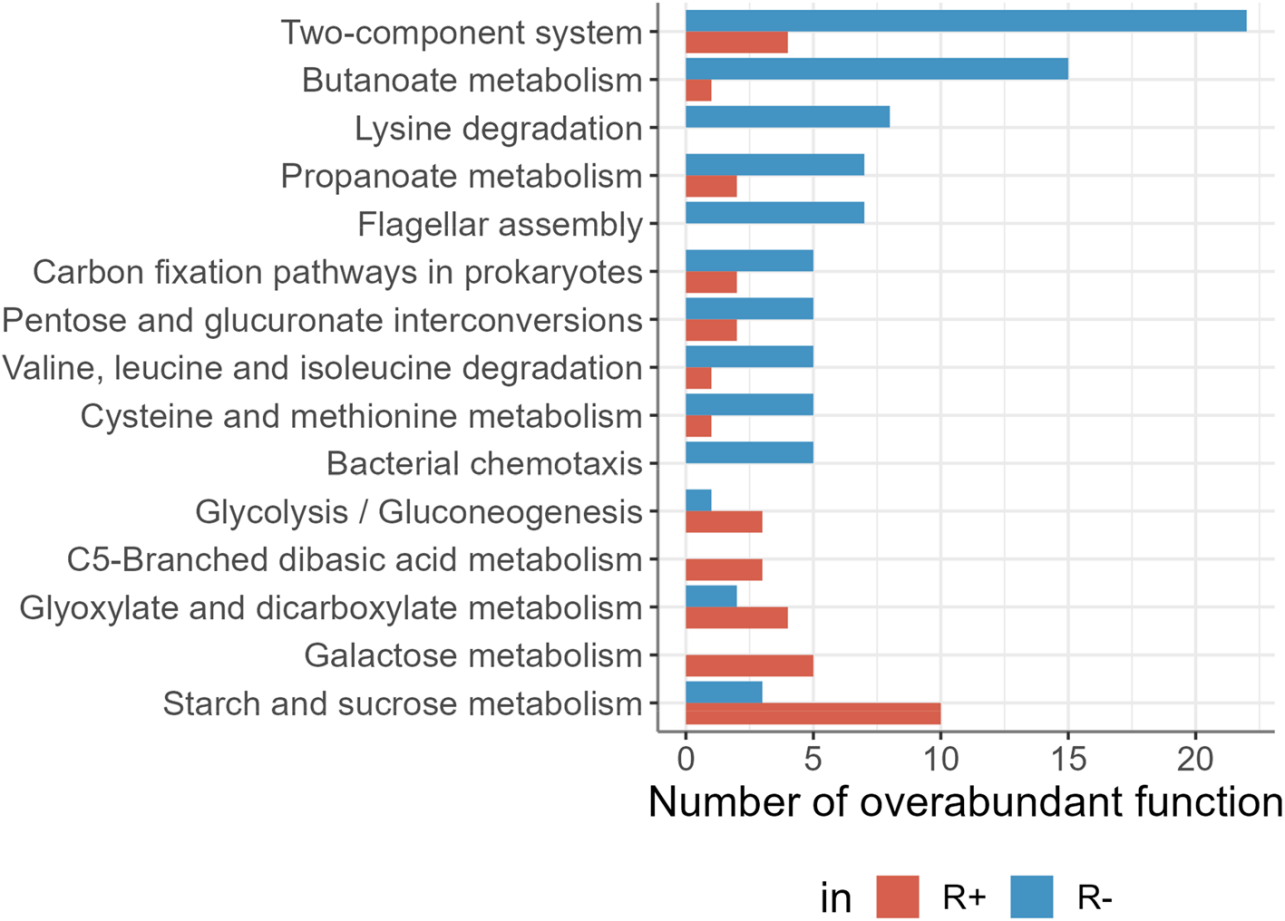
Pathway enriched in differentially abundant functions. Representation of the top 15 represented pathways associated with at least three DA functions. The R+ CTR group is depicted in red and in the R− CTR group in blue.

The DA functions identified in the R+ CTR versus R- CTR comparison could be associated with a large number of pathways, some more abundant in R+ line and others in the R- line (Fig. 4, and supplementary Table S3). In particular, functions more abundant in the R- CTR microbiota were associated with various amino acid metabolisms and degradations, as well as butanoate and propanoate metabolisms. In contrast, the R+ CTR microbiota appeared to be enriched in the metabolism of various carbohydrates, notably starch and sucrose, and galactose.

The similarity between the R− CTR group and the R+ LE group, already observed at the level of DA ASVs, is also observed at functional level. Indeed, pathways that appeared to be more active in the R− CTR group were often also more active in the R+ LE group. On the other hand, pathways related to carbohydrates metabolisms, including starch and sucrose metabolism, which were over-represented in the R+ CTR group, were specific to this line and diet condition.

### Line and diet effects on relative concentrations of microbial short chain fatty acids (SCFA)

The quantification of acetate, propionate, and butyrate revealed that acetate was the most abundant SCFA, accounting on average for 67% of the total SCFA concentration, followed by butyrate (18%) and propionate (15%) (Table 3 and supplementary Figure S1).

**Table 3:** Mean and standard deviation of SCFA relative concentration and significance of line and diet effects.

SCFA	R− CTR	R− LE	R+ CTR	R+ LE	ANOVA *P*-values		
					Line	Diet	Line x Diet
Acetate (%)	64.9 ± 4.2^a^	68.2 ± 3.5^a^	66.7 ± 4.0^a^	68.7 ± 3.5^a^	0.500	0.053	0.796
Propionate (%)	16.7 ± 4.0^a^	15.1 ± 2.1^a^	12.3 ± 4.3^b^	16.0 ± 3.8^a^	0.142	0.397	0.020*
Butyrate (%)	18.4 ± 6.9^a,b^	16.7 ± 2.2^b^	21.0 ± 4.4^a^	15.2 ± 1.7^b^	0.325	0.010*	0.053

ANOVA *P*-values for line effect, diet effect, and interaction were considered significant if ≤ 0.05 and were highlighted with an asterisk. For each SCFA, the relative abundances with different superscripts differed significantly (*P-*value ≤ 0.05).

No significant differences were detected between groups for acetate. However, variations in propionate and butyrate concentrations were notable within the R+ CTR group. The relative concentration of propionate was lower in the R+ CTR group compared to the other groups. Conversely, the relative concentration of the butyrate was significantly higher in the R+ CTR group compared to R+ LE and R− LE groups. While it also tended to be higher than in the R- CTR group, the substantial variance observed in the latter group precludes definitive conclusions.

**Figure 5:**
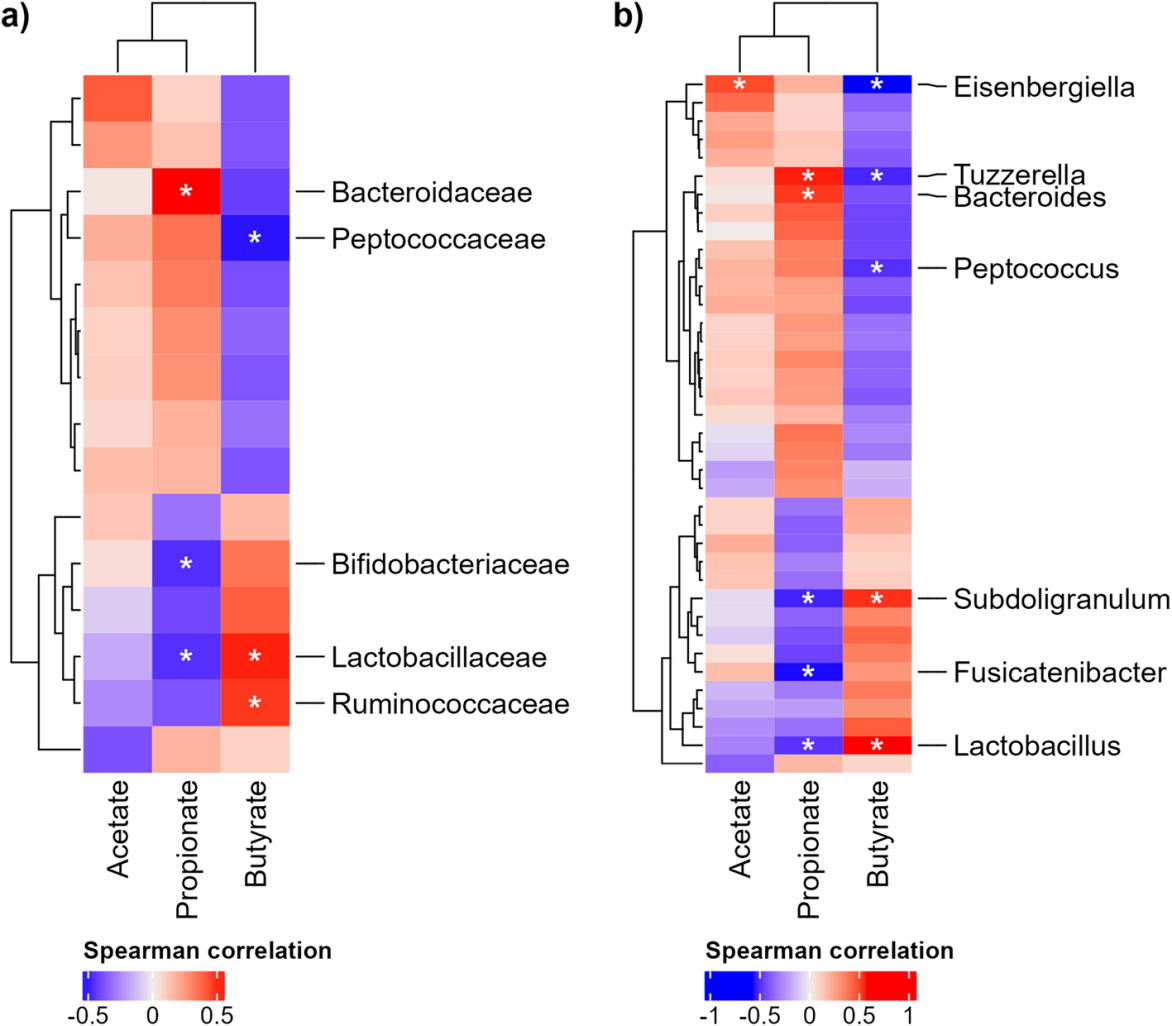
Correlation analysis between relative concentrations of SCFAs and relative abundances of bacterial taxa. (**a**) Correlations at the family level, (**b**) correlations at the genus level. Negative Spearman correlation values below − 0.3 are depicted in blue, while positive values above 0.3 are shown in red. Significant correlations (adjusted *P*-values ≤ 0.05) are indicated with asterisks, along with the associated taxonomies.

Correlation values between the relative concentrations of SCFAs and the relative abundances of families and genera are presented in supplementary Tables S4 and S5, and significant correlations are shown in Fig. 5.

A negative correlation of − 0.6 (*P*-value < 0.001) was observed between propionate and butyrate, as depicted in Fig. 5, where several genera exhibit contrasting correlated values with these two SCFAs. For example, the *Subdoligranulum* and *Lactobacillus* were positively correlated with butyrate, while they were negatively correlated with propionate, and an opposite correlation trend was observed for the genus *Tuzzerella*. Other genera were correlated with only one SCFA. For example, the genus *Bacteroides* was positively correlated with propionate, while *Fusicatenibacter* was negatively correlated.

## Discussion

A better understanding of the role played by the caecal microbiota in chicken feed efficiency could help improve chickens’ ability to extract nutrients from feed. This could have both economic and environmental benefits, including reduction in feed costs and the limitation of feed waste and pollutants.

In this study, we analysed adult laying hens from two experimental lines, one characterised by a low feed efficiency (R+) and the other by a high feed efficiency (R−)^22^. These lines were fed either a standard laying commercial diet (CTR) or a low-energy, high-fibre diet (LE). Previous heritability analyses in these lines have indicated a moderate heritability for RFI (between 0.33 and 0.27)^26^, suggesting that this trait is partly under genetic control and partly controlled by other factors. One such factor could be the influence of the digestive microbiota, which is known to play a role in feed degradation and host metabolism^27^. To explore this aspect, we performed 16S metabarcoding sequencing to characterise the diversity of caecal microbiota in R+ and R− hens across both feeding conditions, and to identify the microbiota functions distinguishing these lines. As in other studies in adult chickens^28–30^ the dominant phyla were *Firmicutes* (45%) and *Bacteroidota* (50%).

### Feed driven changes in microbiota diversity in R+ and R− chicken lines

Our study corroborated previous findings highlighting the significant impact of diet composition on microbiota diversity and richness^21^, in particular emphasizing the central role of fibre content^31^. The LE diet used in this experiment, compared with the CTR diet, was characterised by a 2.1-fold increase in fibre content from various cereal sources, along with a 23% reduction in starch content, resulting in lower metabolisable energy and lower digestibility. Fibres are not digested by the host's digestive enzymes in the small intestine, but undergo partial degradation via fermentation by the caecal microbiota in the large intestine. Fibre composition is of major importance, as soluble fibres in particular, provide a wide range of substrates for fermentation reactions carried out by dedicated microbes, consequently influencing microbiota composition, diversity, and richness^32,33^. In our study, the increase in microbial richness and diversity in hens fed the LE diet is likely due to the wider variety of fibre types in this diet, as the physiochemical properties of different fibres, such as solubility, viscosity, and fermentability, vary considerably depending on their origin^31^. However, this greater variety of fibres affected the R+ and R− lines differently, as shown by the difference in microbial response between the two lines under the LE diet. The microbiota of the efficient R- line was less affected by the change in diet, probably due to its natural tendency for higher microbial richness and a more efficient use of fibre even under the regular CTR diet. This is in line with what reported for the human microbiota, where richer microbiota remained stable despite an increased fibre content in the diet^34^.

In contrast, the microbiota of the R+ line was more affected by the LE diet; the LE formula appeared to drive a dynamic shift that brought the composition of the R+ microbiota closer to that of the R- line. It is highly probable that similar substrates reaching the caecum resulted in similar microbiota compositions between the R+ and R− lines under the LE diet, unlike the scenario observed under the CTR diet.

Interestingly, several ASVs that were more abundant in the R− CTR group were also more abundant in the R+ LE group. This was also observed at the functional level, with numerous functions being more prevalent in both the R− CTR and R+ LE groups. This pattern probably reflects the pressure exerted by the LE diet on the R+ microbiota to expand its functional range in order to degrade the wider variety of substrates offered by a high-fibre regime, activating functions already observed in the R− line under the CTR feeding condition.

### Microbiota richness and diversity differences between efficient and non-efficient hens

A notable finding of our study concerns the genetic effect observed on microbiota richness and diversity, with diet acting as a modulating factor. Particularly, a significant genetic effect was observed under the CTR diet, whereas minimal microbial disparities between efficient and non-efficient hens existed under the LE diet.

The efficiency of digestion and energy harvesting from feed has been associated with the activity and composition of the gut microbiota^35,36^. In this context, the increased caecal microbiota richness observed in the R− line may reflect enhanced nutrient utilisation from feed, potentially contributing to the improved efficiency of R− hens, which are able to maintain production and growth despite a significant reduction in feed intake compared to R+ hens. On the other hand, the specificity of the R+ microbiota under the CTR diet may be related with the higher feed intake characteristic of this line (on average 56% more than R− line). Indeed, ingestion levels and eating patterns have been shown to shape microbiota composition^37^. One hypothesis could be that in the R+ line, excessive feed intake may lead to reduced starch digestibility. While starch is generally well digested by the chicken intestine, some studies have reported a negative correlation between starch digestibility and starch intake, particularly in wheat-based diets^38^. Moreover, wheat is also rich in arabinoxylan, a soluble fibre that can increase viscosity when present in excess and by consequence reduce nutrient digestion^31^. This could result in an overabundance of resistant starch reaching the caecum. Resistant starch is highly fermentable and, like soluble fibres, can modulate microbiota composition. The R+ microbial community might reflect a form of bacterial opportunism prompted by excessive quantities of resistant starch. Indeed, a study on pigs has shown that the composition of the microbiota can be modified in favour of starch metabolism, thereby delaying the fermentation of other soluble fibres like arabinoxylan and β-glucan^39^.

Overall, the observed variations in richness and diversity within the microbiota of efficient and non-efficient hens seem to suggest the presence of two distinct microbial ecosystems. One appears specific to the non-efficient R+ line under the CTR diet, and may be shaped by starch fermentation. The other ecosystem, observed in the efficient R- line under the CTR diet as well as in hens fed the LE diet, is likely capable of degrading a variety of carbohydrates and utilizing a wider range of fibre types as substrates.

### Increased abundance of bacterial clades related to residual starch fermentation in the non-efficient R+ line under the CTR diet

The R+ CTR group is characterised by an abundance of ASVs associated with *Bifidobacterium*. This specificity of *Bifidobacterium* for the R+ CTR group is substantial considering that of the five ASVs associated with this genus, four were not detected in the R- line or in any hen fed the LE diet. We were able to affiliate one of these ASVs to the species *Bifidobacterium pseudolongum* that was particularly abundant in the R+ CTR group (0.5%). Increase in *Bifidobacterium* content was observed often in relation with resistant starch fermentation^40,41^. The starch contained in cereal grains is the main energy source in poultry diets. In the two diets used in this study, starch was 23% lower in the LE diet than CTR diet. Due to the larger feed intake of R+ hens, it is probable that some undigested starch reaches the caecum, leading to starch fermentation. In pigs, this has been correlated with a lower feed efficiency and with an increased population of *Bifidobacterium*^42^, which is in line with our results. The preferential growth of *Bifidobacterium* over other genera during starch fermentation likely occurs because certain strains of *Bifidobacterium* possess the necessary enzymatic activity to utilise starch specifically in the distal gut^43^. Interestingly, we detected increased activity of pathways related to starch and sucrose metabolism in the R+ line under the CTR diet, which supports our hypothesis of differences in the microbial utilisation of starch between the two lines.

Wheat, which largely composes the CTR diet (600g/kg) is also rich in arabinoxylan, a soluble fibre well fermented by the gut microbiota. The fermentation by the microbiota of resistant starch and dietary fibres results mainly in the production of SCFAs, and in the case of resistant starch and arabinoxylan, the fermentation is in favour of the butyrate production^39^. This could explain the observed higher proportion of butyrate produced by the R+ CTR microbiota compared with the other groups.

Butyrate and genus *Bifidobacterium* are generally considered to have beneficial effect on gut health, by improving gut barrier function and maintaining gut homeostasis through cross-feeding interactions with other bacteria^12,44^. Interestingly, the R+ line has been reported to have better immune functions compared to the R- line, presenting lower mortality rate following infection and increased antibody production^3^.

In the R+ CTR group, there were several other ASVs affiliated to SCFA-producing genera, such as *Olsenella* and *Fusicatenibacter*, which are known acetate producers^45,46^ and have previously been associated with resistant starch consumption^47,48^. Numerous ASVs belonging to the genera *Faecalibacterium*, *Subdoligranulum*, and *Fusicatenibacter* (phylum *Firmicutes*) were also more abundant in the R+ CTR group. These genera are known butyrate producers^49,50^ and in our study, the abundance of *Subdoligranulum* was found to be positively correlated with the relative concentration of butyrate. Previous research has identified cross-feeding interactions between *Bifidobacterium* and *Faecalibacterium*^51^, which could explain the abundance of both genera in the R+ CTR group. In another study, *Faecalibacterium* was associated with a low feed efficiency in laying hens^11^, which is consistent with our results.

### Increased abundance of propionate-producing bacteria in the efficient R- line and under LE feeding conditions

Interpreting the patterns of ASVs abundance in the R- line or in the LE diet groups is a complex task, as the majority of the DA ASVs were associated with unknown genera or had multiple affiliations. Nevertheless, among the ASVs that could be associated with known genera, we identified *Anaerosporobacter* as being more abundant in the R- CTR group as well as in the R+ LE group. This genus has been described as cellulose degrader^52^, which may explain its increase in the R+ LE group. Furthermore, it has recently been reported to be negatively correlated with RFI and to contribute to improved feed efficiency in adult layers^53^, in lines with our observation of increase in the R- CTR group. We also identified *Negativibacillus* as being more abundant in both lines in LE diet; this genus being implicated in the digestion of complex carbohydrates^54^.

It also emerged that the large majority of DA ASVs associated with phylum *Bacteroidota* were more abundant in the R− CTR group and/or in the R+ LE group. The role of these bacteria as propionate producers is corroborated by the presence in this phylum of enzymes involved in the succinate pathway, which is the main route for propionate production from ingested carbohydrates^55^. For example, two DA ASVs more abundant in the R- CTR than in the R+ CTR group were associated with genus *Alistipes*. Previous research has linked this genus with improved digestibility^18^ and feed efficiency in broiler chickens and pigs^56–58^. Our results in laying hens are consistent with these earlier findings and support the hypothesis that *Alistipes* may play a role in improving feed efficiency. The *Alistipes* genus is known to degrade cellulose^59^, yet it was not detected as more abundant in the LE diet groups, which is surprising. This observed lack of increase in *Alistipes* abundance under the LE diet may indicate a competitive dynamic among cellulolytic bacteria for the same substrate that will favour the more abundant ones^32^. Indeed, *Bacteroides*, also known to degrade cellulose and to produce propionate^60^, were more abundant than *Alistipes* in the CTR groups and they increased significantly in the R+ LE group. They were also positively correlated with the propionate relative concentration, which was higher in the LE groups.

The potential role of propionate-producing bacteria in enhancing feed efficiency is further supported by the functional analysis, which revealed an activation of the propanoate metabolism pathway in the R− CTR group. This finding is corroborated by the higher relative concentration of propionate in this group compared to the R+ CTR group. This evidence supports the hypothesis that propionate could represent an extra energy source for the host, potentially contributing to improve feed efficiency. Notably, propionate in the liver acts as a precursor for hepatic gluconeogenesis^61^, a process found to be enhanced in the R- line compared to the R+ line, as revealed by the overexpression of key gluconeogenesis genes (Jehl et al. in preparation). It is highly plausible that the additional energy derived from microbial propionate supports the nutritional needs of the R− line, helping to compensate for its greatly reduced feed intake, allowing it to maintain metabolic energy homeostasis.

## Conclusion

This study provides a clear demonstration of the usefulness of the R+ and R− experimental chicken lines, which diverge dramatically with respect to RFI, as models for investigating the role of the gut microbiota in shaping feed efficiency under different feeding conditions. Our results are consistent with the idea that a richer and more diverse microbiota is associated with improvements in hen feed efficiency. It is likely that the long-term regime of intensive selection on high or low RFI values has contributed, either directly or indirectly, to the observed differentiation through the establishment of line-specific gut ecosystems. However, this microbial differentiation between lines could only be detected under the optimal feeding conditions used for the RFI-divergent selection, indicating that the association between the microbiota and feed efficiency is highly feed-dependent. Additionally, we observed that certain taxa and/or inferred functions were shared between the R− CTR group and both LE diet groups, suggesting the existence of common microbiota mechanisms to optimise energy extraction from feed. One such mechanism may be an increase in the abundance of propionate-producing bacteria in order to provide extra energy to host metabolic functions. These results offer insights into how the feed efficiency of laying hens may be influenced by the gut microbiota and, in turn, how these microbial communities are affected by diet composition.

## Methods

### Ethics statement

The experiment was conducted at the experimental farm PEAT (INRAE, Val-de-Loire Center, Nouzilly) under licence number C37-175-1 for animal experimentation, in compliance with European Union Legislation, and was approved by the local ethics committee for animal experimentation (Val de Loire) and by the French Ministries of Higher Education and Scientific Research, and of Agriculture and Fisheries (n°2873-2015112512076871). The study followed the ARRIVE guidelines.

### Animal information and sample collection

This study involved hens from two chicken lines that have been divergently selected for residual feed intake (RFI). RFI represents the deviation of the observed feed intake (FI) of an animal from its predicted feed intake (PFI) calculated based on maintenance and production requirements. These two experimental lines were derived from the same population of Rhode Island Red chickens, and have been developed by the French National Research Institute for Agriculture, Food, and the Environment (INRAE) since 1976 as described in Bordas et al.^22^. This divergent selection program has resulted in an efficient line, named R-, which eats less than estimated (RFI < 0), and an inefficient line, named R+, which eats more than estimated (RFI > 0) despite no differences in terms of growth or eggs production. From this cohort, the present study focused on 58 hens fed two distinct diets. All hens used in this experiment shared the same environment from hatching to sample collection at 31 weeks of age. All eggs were hatched the same day in the same hatchery and chicks were reared together in floor pens under standard rearing conditions until 17 weeks of age. Then, they were transferred into individual cages, with a light regimen set at 14 h of light per day and ad libitum feeding. Of the 58 hens used in this study, 18 hens per line were fed a commercial diet (control group, CTR) and 11 per line were fed a low-energy diet (low-energy group, LE) until they reached 31 weeks of age. At that point, the hens were fed, subjected to head electrical stunning, and immediately slaughtered by neck cut and bleeding. Caecal content was collected shortly after, and samples were snap-frozen in liquid nitrogen and conserved at – 80 °C until DNA extraction was performed.

The two diet formulas, detailed in Table 4, contained similar protein content but differed in energy content. The LE diet was 15% lower in metabolisable energy compared to the CTR diet (9.7 MJ/kg vs. 11.3 MJ/kg). Additionally the LE diet had 23% lower starch content (302 g vs. 393 g) but was 2.1 times higher in raw cellulose (63.2 g/kg vs. 29.6 g/kg). This was due to the reduction of wheat and soybean and to the increase of corn, sunflower, rapeseed, and oat. Metabolisable energy, protein and starch content were confirmed by chemical analyses, and cellulose, hemicellulose and lignin were estimated using Van Soest formulas^62^.

**Table 4:** Composition of CTR and LE diets.

	Diets	
	CTR	LE
Ingredients (g/kg)		
Wheat	599.0	100.0
Corn	50.0	332.5
Cereal co-products	0.0	80.0
Soybean meal	197.6	92.1
Sunflower meal	0.0	152.9
Rapeseed meal	0.0	50.0
Rapeseed seeds	20.0	0.0
Oat	0.0	55.7
Corn gluten meal	2.0	0.0
Alfalfa protein concentrate	5.0	0.0
Soybean oil	10.8	20.0
Minerals, vitamins and pigments	113.0	112.0
Lysine HCl	1.0	1.5
DL-Methionine	0.6	1.1
L-Threonine	0.0	0.2
Energy and nutrient contents		
Metabolisable energy (MJ/kg)	11.3	9.7
Protein (g/kg)	165.0	158.0
Ca (g/kg)	40.0	40.0
Available P (g/kg)	3.4	3.0
Lysine (%)	0.9	0.8
Methionine (%)	0.4	0.4
Starch (g/kg)	393.0	302.0
Cellulose*	29.6	63.2
Hemicellulose*	73.2	95.7
Lignin*	8.9	26.4

CTR correspond to the control diet, and LE to the low-energy, high-fibre diet. Asterisks indicated estimated values using the Van Soest formulas^62^.

### Phenotypic trait collection

Individual traits related to feed efficiency, egg production, and growth were recorded for the 58 hens of the study. Egg number was recorded from 21 to 31 weeks of age and the egg laying rate was estimated as (number of eggs/recorded period)*100. Individual feed intake (FI) was recorded over a four-week period from 28 to 31 weeks of age. Energy intake was calculated by multiplying the FI (g/28 days) by the metabolisable energy of the diets (11.3 MJ/kg and 9.7 MJ/kg for the CTR and LE diets, respectively (Table 4)). Body weight was recorded at 28 (BW28) and 31 weeks (BW31) of age and the body weight gain estimated as BW31–BW28. Egg mass was estimated by adding up the weight of eggs laid over the 28 days recording period. RFI was calculated as the difference between the observed FI and the predicted FI (PFI) for the recorded period: (RFI = FI − PFI). PFI was estimated by a multiple regression equation calculated for all hens using three independent variables: average body weight (BW), BW gain, and egg mass produced over the recorded period^22^. The feed conversion ratio was estimated between 28 and 31 weeks of age as the ratio between the total feed intake and the egg weight produced over that period. Yolk weight was estimated from three eggs per hen collected at week 31 and yolk proportion estimated as (average yolk weight/average egg weight)*100.

### Short-chain fatty acids (SCFAs) in caecal samples

The SCFA (acetate, propionate, and butyrate) content of 40 caecal samples (10 from R− CTR, 10 from R− LE, 11 from R+ CTR, and 9 from R+ LE) was determined by gas chromatography following the protocol previously described by Bedu-Ferrari et al.^63^. Between 100 and 250 mg of caecal content were diluted in two volumes of deionised water. Samples were homogenised and mixed for 2 h at 4 °C before centrifugation at 12,000 × *g* for 15 min at 4 °C. The supernatant was then collected and weighed, and 10% (vol/vol) of phosphotungstic acid saturated solution (Sigma-Aldrich) was added for protein precipitation overnight at 4 °C. As an internal standard, 10 µL of 2-ethylbutyrate (Sigma-Aldrich) were added to 40 µL of acidified supernatant, and the solution was analysed using a gas–liquid chromatograph (GC-FID Agilent 7890B). All samples were analysed in duplicate. Data were collected and peaks were integrated using Agilent OpenLab Chemstation software. Prior to modelling, the relative concentrations of the three SCFAs were computed as the SCFA concentration (in µmol/g) divided by the total concentration of the three main SCFAs (acetate, butyrate, and propionate).

### Metabarcoding sequencing

DNA was extracted from frozen caecal content following the protocol described by Gordon et al.^64^ at the @bridge platform (INRAE, Ile-de-France, Jouy-en-Josas). Subsequently, the V3–V4 hyper-variable regions of the 16S rRNA gene were amplified with two rounds of PCR following the protocol described in Lluch et al.^65^ with the modified primers PCR1F_460 (ACGGRAGGCAGCAG) and PCR1R_460 (TACCAGGGTATCTAATCCT)^66^. Paired-end sequencing (2 × 250 pb) was performed on the Illumina MiSeq Platform.

### Sequence analysis

After removing one sample because of low numbers of raw reads (1108 raw pairs), the sequences of the remaining 57 samples were analysed with FROGS^67^ (version 3.2.3) following up-to-date pipeline guidelines (http://frogs.toulouse.inra.fr/). The specific settings used for the tools were as follows: (i) for read pair assembling with Pear^68^ (version 0.9.10), amplicon size range was set between 300 and 490 bp, (ii) for clustering, the *-d1-fastidious* options were used, (iii) after removing chimeras, we kept clusters with a minimum relative abundance of 0.005% (i.e., more than 96 sequences across all 57 samples), as suggested by Bokulich et al.^69^ and present in at least 30% in at least one all line x diet group, and (iv) for taxonomic affiliation we used the 16S SILVA database (version 138.1)^70^ with a minimal pintail score of 50. Functional analysis was also performed using FROGS (version 4.0.1), which relies on the PICRUSt2 suite (version 2.4.1)^71^. FROGS analysis provides several metrics including the Nearest Sequenced Taxon Index (NSTI) and the percentage of identity and coverage of ASVs on sequences in the PICRUSt2 reference tree. Based on these metrics, we retained only ASVs with NSTI < 0.31, and with an identity and coverage percentage >  = 90% (Supplementary Fig. S2). The remaining ASVs were analysed using the complete pipeline with default parameters to obtain information on KEGG function abundance.

## Statistical analyses

All statistical analyses were performed using R (version 4.1.3), and the significance threshold was set to 0.05.

Microbiota analyses relied on the R packages Phyloseq^72^ (version 1.38), and vegan^73^ (version 2.6.2). All analyses except for those of differential abundance were performed on rarefied counts using the smallest sample abundance (i.e., 14,548 sequences). Alpha diversity was estimated with observed richness and the Shannon index, and beta diversity with Bray–Curtis distance. For the beta diversity analysis, because a betadisper test (package vegan) revealed non-homogeneity of dispersion in the line x diet groups, the impacts of line, diet, and their interaction on distance matrices were tested using the anova.cca test (vegan package) on dbRDA results (capscale function from vegan package using Lingoes adjustment). Sample dissimilarities were visualised using a multidimensional scaling (MDS) ordination plot.

For all other univariate analyses (production and efficiency traits, alpha diversities, phylum relative abundance), a linear model with line, diet, and their interaction as main effects was fitted using the lm function, and Wald Chi-square tests for fixed effects were estimated using the Anova function of the package car^74^(version 3.1-0). If the interaction was not significant, the model was updated with only the line and the diet as fixed effects; instead, when it was significant, the variance analysis was followed by a post-hoc test (package emmeans^75^, version 1.7.5).

For the comparison of SCFA relative concentrations, the same linear model was tested with permutations because of the lack of normality for butyrate, using the lmp function from the lmPerm package^76^ (with *perm* = *"Prob"* and *Ca* = *0.01* options) and the anova function, followed by pairwise line x diet group Wilcoxon tests.

To identify ASVs that were differentially abundant between lines in each diet or between diets in each line, we used the DESeq2^77^ package (version 1.34) on non-rarefied ASV abundances, with the poscounts estimate size factors method, and Benjamini Hochberg *P*-value adjustment. DESeq2 analyses were also performed on abundance tables of KEGG functions between diets in each line and between lines in each diet, with default parameters but with the more-conservative Benjamini Yekutieli *P*-value adjustment method. Differentially abundant functions were visualised using an Ipath3 metabolic pathways map^78^.

Finally, Spearman correlation analysis was performed between the relative concentrations of SCFAs and the relative abundance of bacterial families or genera using the corr.test function from the psych package^79^(version 2.2.9) (with *adjust* = *"BH", use* = *"complete"* options). Correlation results were visualised using ComplexHeatmap package^80^ (version 2.10.0) with default clustering options of rows (families or genera) and column (SCFA), i.e. complete method on Euclidean distance.

### Supplementary Information

Supplementary Figures.

Supplementary Tables.

## Data Availability

The sequences generated and analysed during the current study are available in the PRJEB62837 repository, https://www.ebi.ac.uk/ena/browser/view/PRJEB62837.
