# Supplementary material for: Relationship between feed efficiency and gut microbiota in laying chickens under contrasting feeding conditions
Source: Sci Rep. 2024 Apr 8;14:8210. doi: 10.1038/s41598-024-58374-3 (PMC11001975; doi:10.1038/s41598-024-58374-3)
Supplement: Supplementary file 1 — Supplementary Figures. [file 41598_2024_58374_MOESM1_ESM.docx]

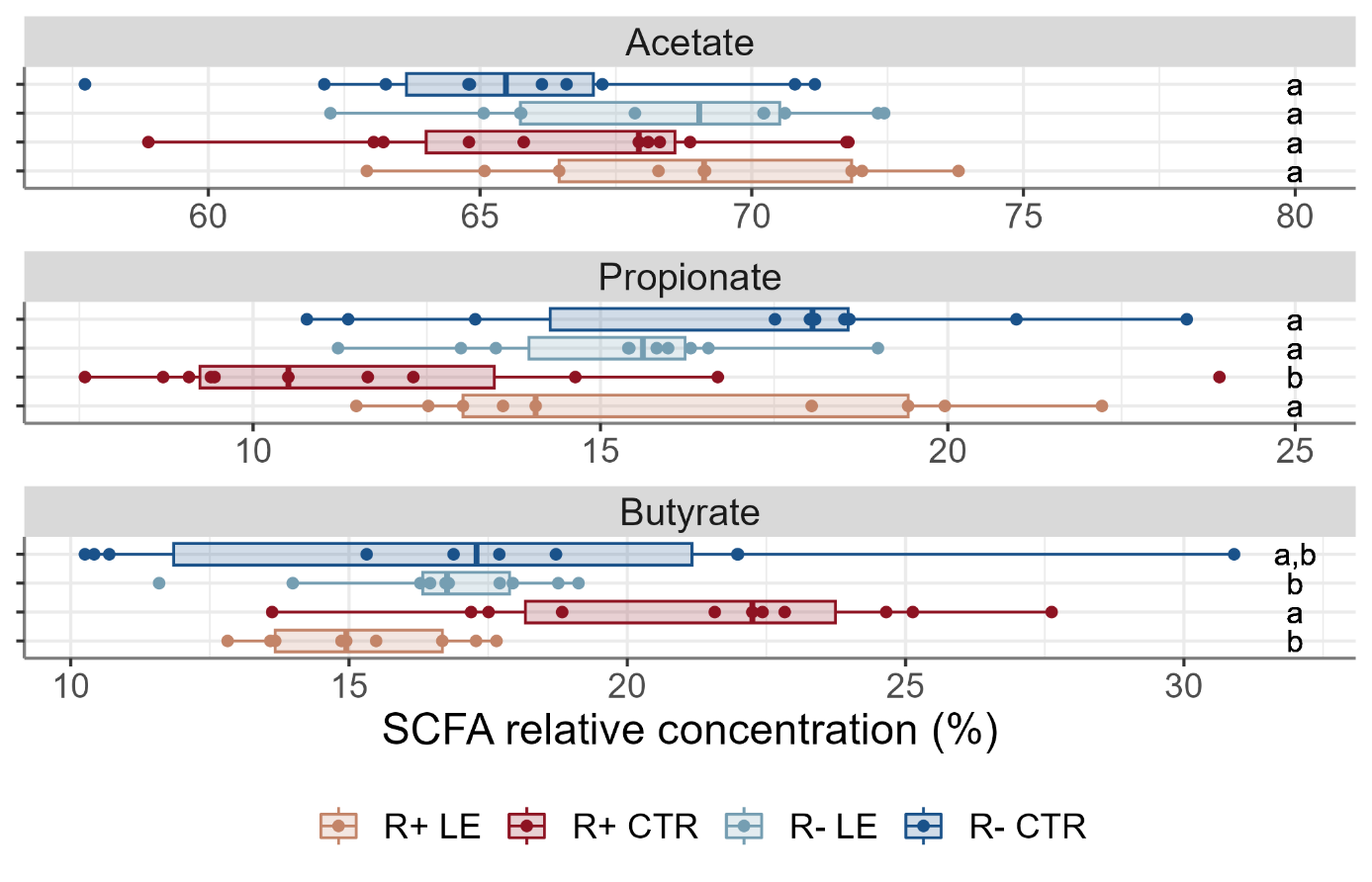


**Supplementary Figure S1 : Distribution of relative SCFA concentration.** Distribution of the relative SCFA concentration (%) within each line x diet group: red= R+ line fed the CTR diet; blue= R- line fed the CTR diet; orange= R+ line fed the LE diet; light blue= R- line fed the LE diet. Different letters at the top indicate significant differences from Wilcoxon pairwise tests;


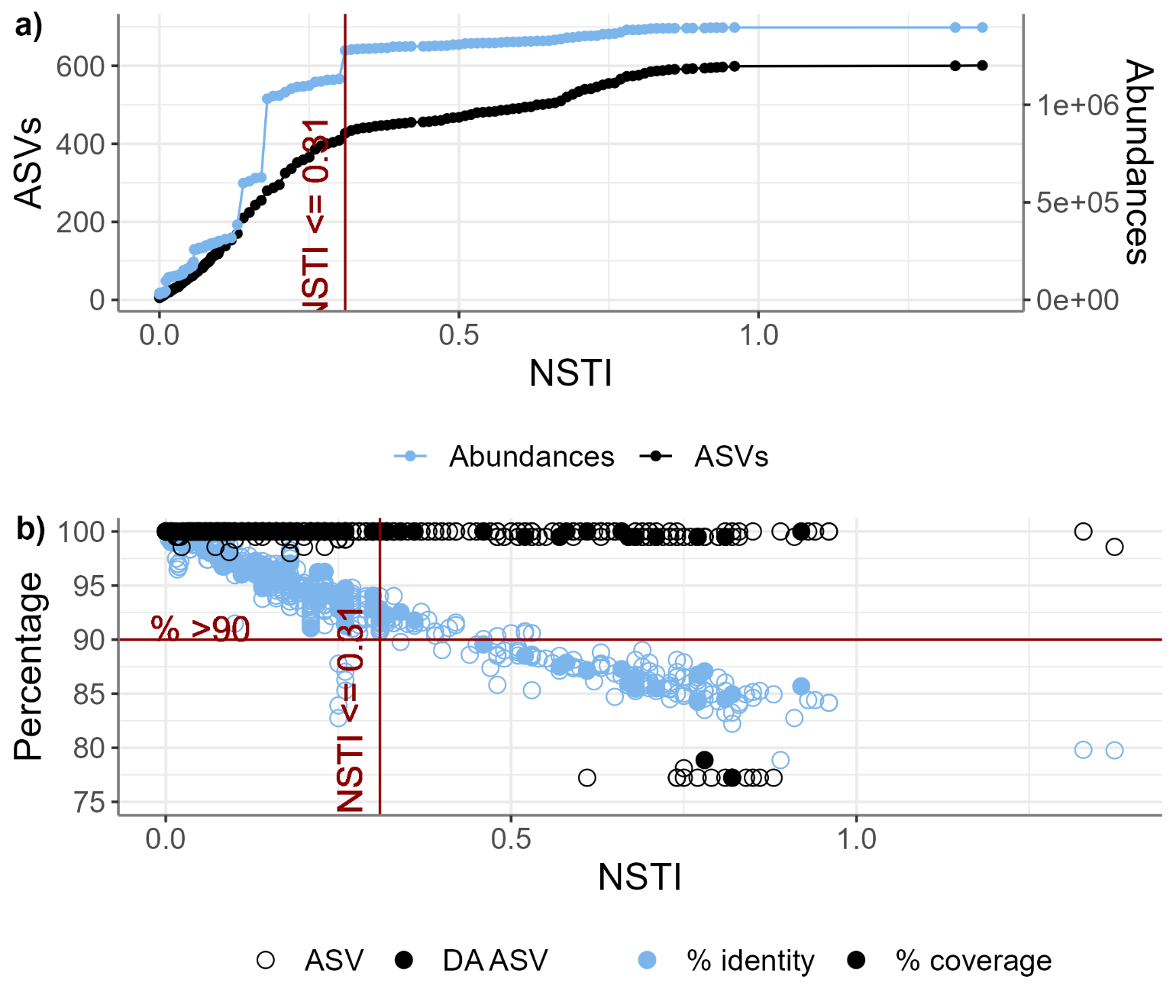


**Supplementary Figure S2: Nearest Sequence Tree Index (NSTI) distribution as a function of identity percentage and alignment coverage.** a) Number of ASVs and associated abundances bellow the chosen NSTI threshold; b) Distribution of differentially (solid circle) or not (empty circle) ASVs as a function of its NSTI value, identity and alignment coverage percentage.
